# Baseline podocyte‐associated state and persistent cell–matrix transcriptional programs are associated with BALB/c substrain differences in adriamycin nephropathy

**DOI:** 10.1002/ame2.70281

**Published:** 2026-08-24

**Authors:** Ryuya Nakagawa, Masaki Watanabe, Koyuki Kawamoto, Momoka Hiratsuka, Takeru Sasaki, Nobuya Sasaki

**Affiliations:** ^1^ Laboratory of Laboratory Animal Science and Medicine, School of Veterinary Medicine Kitasato University Towada Aomori Japan

**Keywords:** Adriamycin nephropathy, BALB/c substrain, extracellular matrix remodeling, podocyte injury, transcriptomics

## Abstract

**Background:**

Adriamycin (ADR)‐induced nephropathy is a widely used murine model of glomerular injury. Although strain‐dependent susceptibility is well recognized, variation among closely related substrains remains insufficiently explored.

**Methods:**

We compared early responses to ADR between two BALB/c substrains, BALB/cAJcl (AJcl) and BALB/cByJcl (ByJcl), focusing on baseline podocyte‐associated features and early glomerular transcriptional programs.

**Results:**

Following ADR administration, ByJcl mice developed significantly greater albuminuria at Day 7 compared with AJcl mice. At Day 7, ByJcl mice also showed lower WT1‐positive nuclei counts and diminished nephrin immunoreactivity than AJcl mice; notably, these differences were already evident at baseline. Glomerulus‐enriched RNA sequencing showed that extracellular matrix organization and integrin signaling were already enriched in ByJcl at baseline and remained enriched at Day 5, when coordinated suppression of podocyte‐associated transcriptional programs was also evident.

**Conclusion:**

Substrain‐dependent differences in baseline podocyte‐associated readouts and a pre‐existing cell–matrix transcriptional state that persists after ADR are associated with divergent trajectories of ADR‐induced injury. Because WT1‐based counts cannot distinguish podocyte number from WT1 expression, these baseline differences are best described as an attenuated podocyte‐associated state rather than a definitive reduction in podocyte number. Careful documentation of substrain origin is critical for experimental reproducibility in nephropathy research.

## Graphical abstract

Two closely related BALB/c substrains exhibit different early responses to adriamycin (ADR)‐induced nephropathy. BALB/cByJcl mice showed lower baseline WT1‐positive nuclei counts and NPHS1 immunoreactivity than BALB/cAJcl mice, together with extracellular matrix and integrin programs that were enriched at baseline and persisted at Day 5. At Day 5, podocyte‐associated transcriptional programs were relatively attenuated in BALB/cByJcl mice, which developed greater albuminuria and more severe early renal injury at Day 7. These findings associate pre‐existing podocyte‐associated and cell‐matrix states with divergent ADR injury trajectories and underscore the importance of substrain documentation.

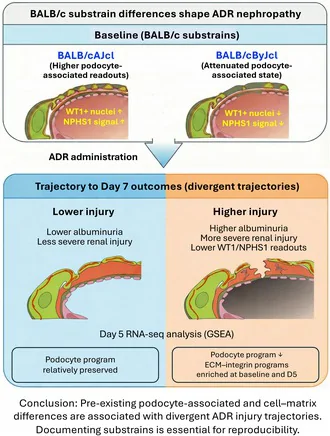

## 1 INTRODUCTION

Adriamycin (ADR; doxorubicin)‐induced nephropathy is one of the most widely used murine models of glomerular injury and is considered to recapitulate key pathological features of human focal segmental glomerulosclerosis (FSGS), including podocyte injury, albuminuria, and progressive glomerulosclerosis.^1^ Following a single systemic administration of ADR, susceptible mouse strains develop early podocyte damage characterized by reduced slit diaphragm integrity, cytoskeletal disorganization, and impairment of the glomerular filtration barrier. As injury progresses, secondary tubular stress responses and interstitial changes may arise and contribute to overall disease severity.^2^, ^3^ Owing to its reproducibility and pronounced strain‐dependent variability, the ADR model has been extensively used to investigate genetic determinants of glomerular vulnerability and mechanisms of podocyte injury.

Genetic background is a major determinant of susceptibility to ADR‐induced nephropathy. Marked differences among inbred strains—such as BALB/c and C57BL/6—have been well documented with respect to albuminuria severity, glomerulosclerosis, and survival, underscoring the importance of host genetic modifiers in shaping disease progression.^4^, ^5^, ^6^ In contrast, comparatively less attention has been paid to variation among closely related substrains, and substrain information is often underreported in practice. Nevertheless, long‐term separation, genetic drift, spontaneous mutations, and colony‐specific selection pressures can generate biologically meaningful divergence over time. Indeed, substrain‐dependent differences in ADR susceptibility have been reported even within the commonly used C57BL/6 background: C57BL/6N exhibits susceptibility to ADR‐induced glomerular injury, whereas C57BL/6J is relatively resistant, and additional variability has been observed among C57BL/6N substrains.^7^, ^8^

The BALB/c strain is widely used in nephropathy research and is generally regarded as susceptible to ADR‐induced glomerular injury.^9^ Nevertheless, multiple BALB/c substrains are maintained independently by different breeders, and their molecular and pathological equivalence cannot be assumed. Consistent with this, phenotypic divergence among BALB/c substrains has been reported in other biological contexts, including immune‐related traits, indicating that independent colony maintenance can lead to measurable functional differences.^10^, ^11^ Even subtle divergence may influence the baseline state of podocytes—such as podocyte endowment per glomerulus and/or the expression of slit diaphragm components—thereby modulating the extent of injury amplification following ADR exposure. Defining such baseline predisposition is important not only for mechanistic interpretation but also for experimental reproducibility and cross‐study comparability.

In addition to baseline cellular context, early injury‐response programs within the glomerulus may shape divergent trajectories after ADR exposure. Podocyte stability critically depends on interactions with the glomerular basement membrane (GBM), which are mediated in part by integrin‐dependent adhesion and signaling.^12^ Perturbation of integrin–GBM interactions can promote podocyte detachment and proteinuria, and integrin‐dependent pathways are increasingly recognized as central regulators of glomerular cell behavior and matrix organization in health and disease.^13^ In parallel, extracellular matrix (ECM) programs—including collagen‐related processes and matrix remodeling—can alter GBM composition and biomechanical properties.^14^ Importantly, activation of ECM–adhesion pathways during early injury may reflect a range of biological states, including compensatory remodeling, early maladaptive responses, or shifts in glomerular cellular composition. Therefore, characterizing these programs at early time points may provide insight into determinants of susceptibility before overt histological separation becomes apparent.^12^, ^14^

Whether closely related BALB/c substrains differ in these baseline and early injury‐response features remains unclear. In the present study, we therefore compared two ADR‐susceptible BALB/c substrains, BALB/cAJcl (AJcl) and BALB/cByJcl (ByJcl)—both available from a single domestic vendor (CLEA Japan), allowing them to be sourced, housed, and acclimatized under identical conditions and thereby minimizing vendor‐ and environment‐related confounding—with respect to early responses to ADR administration. We assessed semi‐quantitative urinary albumin‐to‐creatinine ratio (Alb/Cre), tubular cast formation, and podocyte integrity using WT1‐positive cell counts and nephrin (NPHS1) immunoreactivity, together with a tubular injury marker. In addition, we performed glomerulus‐enriched transcriptomic profiling at baseline and at an early post‐ADR time point preceding overt morphological separation. We hypothesized that substrain‐dependent differences in baseline podocyte‐associated state and cell–matrix transcriptional programs, together with their persistence during early ADR injury, contribute to differential susceptibility to ADR‐induced nephropathy.

## 2 METHODS

### 2.1 Animals

Eight‐week‐old male BALB/cAJcl and BALB/cByJcl mice were obtained from CLEA Japan (Tokyo, Japan). Mice were maintained under specific pathogen‐free conditions in a temperature‐controlled facility (22 ± 2°C) on a 12‐h light/dark cycle, with ad libitum access to standard laboratory chow and water. All procedures followed the Regulations for Animal Experiments of Kitasato University. The protocol was reviewed by the Kitasato University Institutional Animal Care and Use Committee and approved by the University President (Approval ID: 24–094). Humane endpoints required marked body‐weight loss (≥20%) together with severe systemic deterioration (e.g., generalized edema, anemia, or markedly reduced activity); weight loss alone was not an endpoint. No animal reached 20% body‐weight loss, met the humane‐endpoint criteria, or was euthanized before the scheduled endpoint or lost. No animal was excluded from the in vivo study; one RNA‐seq library was excluded from the primary transcriptomic analysis based on the quality‐control considerations described below.

### 2.2 Adriamycin‐induced nephropathy

Adriamycin (doxorubicin hydrochloride; Fujifilm Wako Pure Chemical Corp., Osaka, Japan) was freshly dissolved in sterile saline at 2 mg/mL, protected from light, and administered at 10 mg/kg (a volume of 5 μL/g body weight) as a single dose via retro‐orbital injection; control mice received the same volume of sterile saline. The retro‐orbital route was used because we have previously validated it for the BALB/c ADR nephropathy model, where 10 mg/kg by this route produced albuminuria, tubular injury, and renal fibrosis indistinguishable from conventional tail‐vein administration while reducing restraint and stress.^15^ The injection was performed under isoflurane anesthesia with mice placed in lateral recumbency and the injected eye facing upward. After gentle proptosis, a 29‐gauge needle was inserted at the medial canthus along the lower margin of the globe at an approximately 30° angle, and the ADR solution was injected slowly. The needle was then withdrawn carefully. Mice were assigned to treatment groups within each substrain without formal randomization; all animals were age‐ and sex‐matched (8‐week‐old males) and, at study onset (Day 0), body weights ranged from 27.4 to 33.5 g. Body weight and general condition were monitored daily. Transcriptomic and histological/urinary analyses were performed on separate cohorts: one cohort was used for glomerulus‐enriched transcriptomic analysis, with kidneys harvested on Day 5 (D5), and a separate cohort was used for functional and histological evaluation, with spot urine collected and kidneys harvested on Day 7 (D7). Within the Day 7 cohort, urine, histology, and body‐weight data were obtained from the same animals. Day 5 was selected for transcriptomic profiling, and Day 7 for histological and functional assessment, because in adriamycin nephropathy molecular and cellular changes precede overt structural and functional injury. Profiling at Day 5 was therefore intended to capture early transcriptional programs before secondary structural remodeling could confound the signal, whereas Day 7 provided an appropriate window to quantify the resulting functional (albuminuria) and histological changes.

### 2.3 Urinary albumin assessment by SDS–PAGE and Alb/Cre calculation

Urine was collected on Day 7 after ADR administration. Samples were obtained from the urinary bladder under anesthesia and clarified by centrifugation to remove debris. Urinary albumin was assessed by SDS–PAGE using a method consistent with our prior work.^16^ This approach was selected because it allows direct visualization of the albumin‐sized urinary protein band and provides molecular‐weight‐based information on urinary protein patterns. In addition, SDS–PAGE‐based detection is not dependent on antibody recognition and may therefore complement antibody‐based albumin assays, which can be affected by species specificity or altered immunoreactivity of urinary albumin species.^17^ Briefly, equal volumes of urine were mixed with sample buffer and electrophoresed on polyacrylamide gels, with bovine serum albumin (2.5 μg) run in parallel as a positive control. After electrophoresis, gels were stained with Coomassie Brilliant Blue using a Rapid Stain CBB kit (Nacalai Tesque, Kyoto, Japan) and destained in distilled water. Gel images were captured using a flatbed scanner (GTX‐820, EPSON, Nagano, Japan). A representative Coomassie‐stained gel is provided in Figure S1. For semi‐quantification, the intensity of the albumin band was measured using Fiji/ImageJ (version 1.52a; National Institutes of Health, Bethesda, MD, USA). Urinary creatinine concentrations were determined from the same urine samples using a Creatinine Colorimetric Assay Kit (Cayman Chemical, Ann Arbor, MI, USA) according to the manufacturer's instructions. The albumin‐to‐creatinine ratio (Alb/Cre) was calculated by normalizing albumin band intensity to the corresponding urinary creatinine value. Because this readout is based on densitometric quantification of Coomassie‐stained bands, Alb/Cre was treated as a semi‐quantitative urinary albumin/creatinine index rather than as an ELISA‐equivalent quantitative uACR measurement. Alb/Cre values are expressed as arbitrary units normalized to urinary creatinine concentration (a.u./mg/dL creatinine; a.u., arbitrary units).

### 2.4 Histological evaluation and tubular cast scoring

Kidneys were fixed in 10% neutral buffered formalin, embedded in paraffin, and sectioned at 2 μm for periodic acid–Schiff (PAS) staining. After deparaffinization and rehydration, sections were oxidized in 0.5% periodic acid for 5 min, rinsed in distilled water, and incubated with Schiff's reagent (Fujifilm Wako Pure Chemical Corporation, Osaka, Japan) for 30 min. Sections were then washed three times with 0.8% sodium bisulfite for 3 min each, rinsed in running water, counterstained with hematoxylin for 4 min, and processed through dehydration and clearing before mounting, following established protocols.^18^ Tubular cast formation was evaluated semi‐quantitatively in the renal cortex using the following scoring system: 0: no casts, 1: <10% of tubules, 2: 10%–25%, 3: 25%–50%, 4: >50%. Scoring was performed independently by two blinded observers, and the average value was used for statistical analysis.

### 2.5 Immunohistochemistry

Paraffin sections were deparaffinized and rehydrated. Antigen retrieval was performed by heating sections at 98°C for 45 min in Immunosaver solution (Nissin EM, Tokyo, Japan). Endogenous peroxidase activity was quenched with 3% hydrogen peroxide, followed by blocking with 5% goat serum. Sections were incubated overnight at 4°C with rabbit anti‐nephrin/NPHS1 (1:4000, Proteintech, Rosemont, IL, USA) and rabbit anti‐WT1 (1:1000, Proteintech). After washing, sections were incubated with an HRP‐conjugated secondary antibody (Histofine SimpleStain MAX PO [Rabbit], Nichirei Biosciences, Tokyo, Japan), and signals were visualized using 3,3′‐diaminobenzidine (DAB; ImmPACT DAB Substrate Kit, Vector Laboratories, Newark, CA, USA). Sections were counterstained with hematoxylin, dehydrated, cleared, and mounted.

WT1‐positive nuclei were counted manually in 30 glomerular cross‐sections per mouse (*n* = 4 baseline and *n* = 7 ADR mice per substrain), and the per‐mouse mean was used as the unit of analysis; the mouse was the experimental unit. Slides were prepared by an investigator aware of group allocation, but the investigators who acquired the images and counted WT1‐positive nuclei were blinded to substrain and treatment group. Unlike NPHS1 (below), WT1 produces a discrete nuclear signal that does not form a continuous glomerular outline, precluding reliable automated glomerular segmentation; glomeruli were therefore counted manually.

Nephrin (*Nphs1*) immunoreactivity was quantified from brightfield images using a custom Python‐based pipeline (NumPy, pandas, scikit‐image, SciPy, and PIL). Briefly, RGB images were subjected to HED color deconvolution, and the DAB channel was used to derive a normalized optical‐density metric (scaled to 0–1 using the 1st–99th percentile range). The 1st and 99th percentile values were determined globally across the entire image dataset and applied identically to all images. Images were analyzed at reduced resolution (downsample factor = 4), and candidate glomerular ROIs were detected by Otsu thresholding of a Gaussian‐smoothed DAB optical‐density image (sigma = 2), followed by morphological filtering and removal of border‐touching objects. For each glomerulus, a convex‐hull ROI with a small margin expansion was generated, and the mean DAB optical density within the ROI (Mean_OD; higher values indicate stronger DAB signal) was calculated. Segmentation overlays were visually inspected for quality control; representative accepted glomerular ROI detections are provided in Figure S2. Because NPHS1 produces a continuous membranous DAB signal that reliably delineates the glomerular outline, automated segmentation was considered suitable for this analysis. All accepted glomeruli per sample were analyzed (773 glomeruli across 22 mice; median, 35 per mouse; range, 23–47 per mouse), rather than a fixed random subsample, and the per‐mouse mean Mean_OD across all accepted glomeruli was used for statistical analyses (*n* = 4 baseline and *n* = 7 ADR mice per substrain). NPHS1 optical density was quantified using the automated pipeline with identical parameters applied to all images, thereby minimizing observer‐dependent variation in ROI selection and signal measurement. Segmentation overlays were visually inspected only for quality control.

### 2.6 Glomerular isolation

Glomeruli were isolated on Day 5 using a Dynabeads‐based perfusion method as described by Takemoto et al.^19^ Briefly, mice were perfused via the left ventricle with phosphate‐buffered saline containing magnetic beads (Dynabeads™ M‐450, 4.5 μm diameter; Thermo Fisher Scientific, Waltham, MA, USA), allowing bead entrapment within glomerular capillaries. Kidneys were excised, minced, and digested in collagenase at 37°C. The suspension was passed through a 100‐μm cell strainer, and bead‐containing glomeruli were collected using a magnetic separator and washed repeatedly with PBS. Purity of isolated glomeruli was confirmed by light microscopy prior to RNA extraction.

### 2.7 RNA extraction and sequencing

Total RNA was extracted from isolated glomeruli using the NucleoSpin RNA kit (Takara Bio, Shiga, Japan) according to the manufacturer's instructions. RNA quantity and integrity were evaluated using a NanoDrop spectrophotometer and an Agilent Bioanalyzer, and only samples with an RNA integrity number (RIN) ≥7.0 were used for downstream analyses. RNA‐seq library preparation and sequencing were outsourced to GENEWIZ, Inc. (Tokyo, Japan). Libraries were generated using poly(A) selection for strand‐specific RNA‐seq and sequenced on a NovaSeq 6000 system (Illumina, San Diego, CA, USA) to obtain 2 × 150 bp paired‐end reads (approximately 6 Gb per sample). For primary data processing, raw reads were quality‐checked with FastQC (v0.11.9),^20^ trimmed with Trimmomatic (v0.39),^21^ aligned to the mouse reference genome (*Mus musculus*, GRCm39) using STAR (v2.7.10a),^22^ and summarized at the gene level using featureCounts (Subread v2.0.3).^23^

### 2.8 Differential expression and pathway analysis

Differential expression analysis was performed using DESeq2 (v1.38.3).^24^ All substrain contrasts were computed as ByJcl relative to AJcl, such that a positive log2 fold change indicates higher expression in ByJcl. Genes with a Benjamini–Hochberg adjusted *p* value (FDR) <0.05 were considered differentially expressed. Differential expression tables for all comparisons are provided as Table S1.

To evaluate coordinated pathway‐level shifts, preranked gene set enrichment analysis was conducted using the fgsea package (v1.24.0; multilevel algorithm)^25^ in R, with all detected genes ranked by the DESeq2 Wald statistic. Reactome gene sets were obtained from MSigDB (M2:CP:REACTOME collection).^26^, ^27^ For each comparison, normalized enrichment scores (NES) and Benjamini–Hochberg false discovery rates were computed jointly across all tested gene sets, and gene sets with FDR <0.05 were considered significantly enriched. Podocyte‐associated transcriptional programs were assessed using a podocyte marker gene set derived from single‐cell RNA sequencing of mouse glomeruli,^28^ provided as a GMT file (gene list in Table S2); a more stringent podocyte‐exclusive marker set was used as a confirmatory panel.

In addition, over‐representation analysis of the DESeq2‐derived differentially expressed genes (FDR <0.05) against Reactome pathways was performed using ReactomePA (v1.42.0; enrichPathway),^29^ with all tested genes as the background and Benjamini–Hochberg correction; terms with adjusted *p* < 0.05 were considered significantly enriched. Reactome pathways were defined according to the Reactome knowledgebase.^30^

Because one BALB/cAJcl ADR sample (A‐ADR1) showed reduced glomerular purity during quality control—reflected in the lowest podocyte‐to‐tubular marker ratio among the Day 5 samples and elevated renal‐tubular marker expression (Figure S3)—a sensitivity analysis was performed by repeating the differential expression and enrichment analyses after excluding this sample. Baseline samples formed a continuous distribution on this metric and were all retained. Full A1 include/exclude sensitivity results for the focal gene sets are provided in Table S3.

Complete Day‐5 ByJcl‐versus‐AJcl GSEA and ORA results are provided in Table S4; focal results across the four primary comparisons are provided in Table S5.

### 2.9 Statistical analysis

Continuous data analyzed using parametric tests are presented as mean ± SEM with individual values, unless otherwise stated. Ordinal or non‐normally distributed data are presented as box‐and‐whisker plots with individual values. Comparisons between two substrains for continuous data were performed using Welch's two‐tailed *t*‐test. Ordinal data, including tubular cast scores, were analyzed using the Mann–Whitney *U* test. Pairwise substrain comparisons of WT1‐positive nuclei counts at baseline and Day 7 were also performed using the Mann–Whitney *U* test. To jointly evaluate the effects of substrain and ADR treatment, per‐mouse mean WT1‐positive nuclei counts and NPHS1 optical density were analyzed by two‐way ANOVA with substrain and treatment as fixed factors, followed by Tukey's HSD post hoc test for multiple comparisons where appropriate. All tests were two‐sided, and a *p* value <0.05 was considered statistically significant. Statistical analyses were performed in Python using SciPy, pandas, and statsmodels.

## 3 RESULTS

### 3.1 Early exacerbation of ADR‐induced albuminuria in BALB/cByJcl mice

**FIGURE 1:**
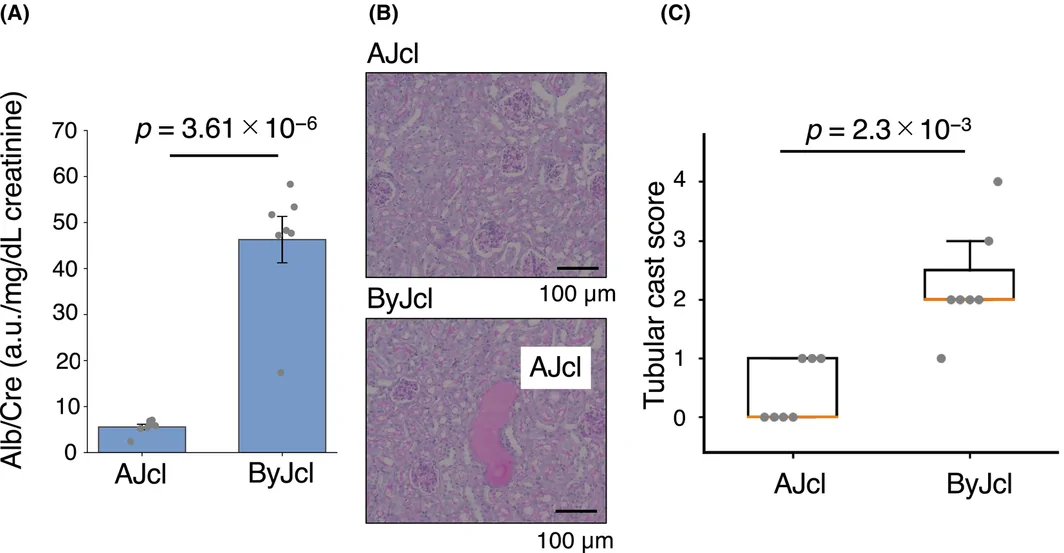
Early exacerbation of ADR‐induced albuminuria and tubular cast formation in BALB/cByJcl mice. (A) Semi‐quantitative urinary albumin‐to‐creatinine ratio (Alb/Cre; albumin band intensity [a.u.] normalized to urinary creatinine concentration) at Day 7 (D7) after a single intravenous injection of adriamycin (ADR) in BALB/cAJcl (AJcl) and BALB/cByJcl (ByJcl) mice (*n* = 7 per substrain). Differences between substrains were evaluated using Welch's two‐tailed *t*‐test. (B) Representative periodic acid–Schiff (PAS)‐stained renal cortical sections at D7 after ADR administration. Scale bar, 100 μm. (C) Semi‐quantitative tubular cast score in the renal cortex at D7 (*n* = 7 per substrain). Casts were scored as 0 (none), 1 (<10% of tubules), 2 (10–25%), 3 (25–50%), or 4 (>50%) by two blinded observers, and the averaged score was used for analysis. Data presentation and statistical tests are described in Methods; statistical significance is indicated in the graphs. Differences between substrains were evaluated using the Mann–Whitney *U* test.

To compare early susceptibility to ADR‐induced nephropathy between BALB/c substrains, AJcl and ByJcl mice were analyzed 7 days after a single administration of ADR. Urinary albumin‐to‐creatinine ratio (Alb/Cre), a functional indicator of glomerular filtration barrier integrity, was significantly higher in ByJcl mice than in AJcl mice at Day 7, indicating more severe early glomerular dysfunction in the ByJcl substrain (Figure 1A).

PAS staining showed no marked difference in overall glomerular architecture at this early time point. We therefore examined tubular cast formation as an early morphological correlate of albuminuria. Casts were more frequently observed in ByJcl kidneys, and semi‐quantitative cast scores were higher in ByJcl than in AJcl. These casts may represent proteinuria‐associated tubular changes accompanying the more severe glomerular phenotype in ByJcl mice, although a contribution from tubular injury cannot be excluded (Figure 1B,C).

Body weight was monitored in ADR‐treated mice through Day 7 to assess whether substrain differences in systemic toxicity might confound the renal phenotype. ADR‐treated AJcl mice lost more weight than ByJcl mice (mean nadir −13.8% vs. −9.8%; Mann–Whitney *p* = 0.017). Two of seven AJcl mice transiently exceeded 15% body‐weight loss, whereas no ByJcl mouse did; however, all animals remained below 20% body‐weight loss, and none met the humane endpoint or was lost or excluded (Figure S4). Thus, ByJcl mice exhibited less body‐weight loss despite greater renal injury, indicating that the renal phenotype was not accompanied by greater systemic weight loss.

### 3.2 Lower podocyte‐associated readouts in ByJcl mice at day 7

**FIGURE 2:**
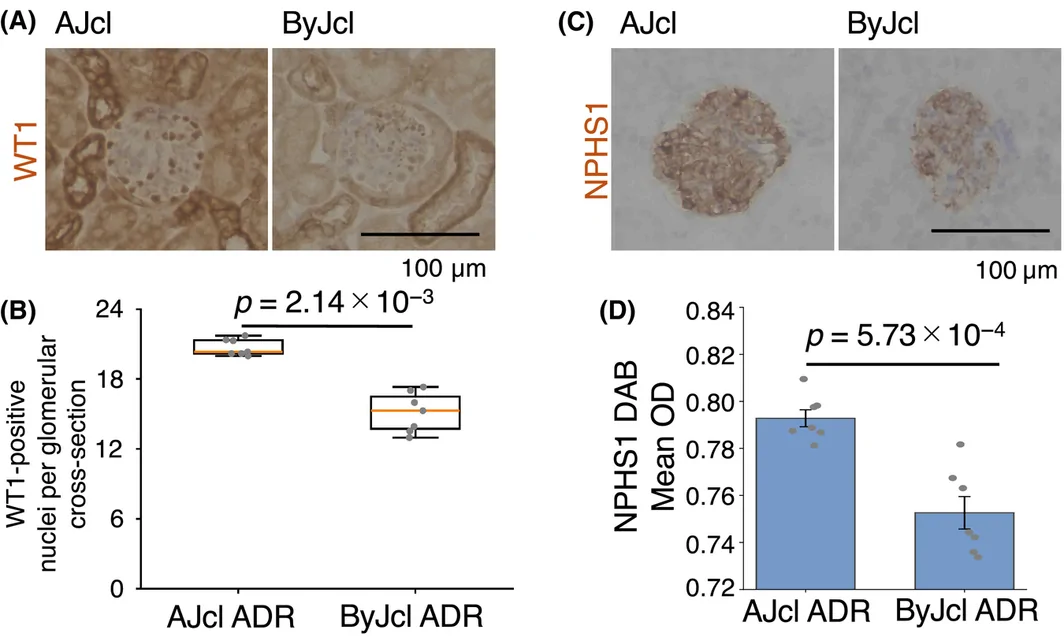
Lower podocyte‐associated readouts in ByJcl glomeruli at Day 7 after ADR. (A) Representative immunohistochemistry for WT1 in glomeruli from AJcl and ByJcl mice at Day 7 (D7) after ADR (*n* = 7 per substrain). (B) WT1‐positive nuclei per glomerular cross‐section (*n* = 7 per substrain; 30 glomerular cross‐sections per mouse; blinded counting; per‐mouse means used for statistics). Differences between substrains were evaluated using the Mann–Whitney *U* test. Data are presented as box‐and‐whisker plots with individual per‐mouse values. (C) Representative immunohistochemistry for nephrin (NPHS1/Nphs1) at D7 after ADR (*n* = 7 per substrain). (D) Glomerular nephrin immunoreactivity, measured as mean DAB optical density (Mean_OD) in automatically segmented accepted glomerular ROIs (*n* = 7 per substrain; all accepted glomeruli quantified; per‐mouse means used for statistics). Differences between substrains were evaluated using Welch's two‐tailed *t*‐test. Data are presented as individual per‐mouse values with mean ± SEM. Scale bars and statistical significance are indicated in the panels.

Given the early divergence in albuminuria, we next examined podocyte‐associated markers, as altered expression of podocyte markers is a hallmark of ADR‐induced glomerular injury. Immunohistochemical analysis revealed that the number of WT1‐positive nuclei per glomerular cross‐section was significantly lower in ByJcl mice than in AJcl mice at Day 7 (Figure 2A,B); as shown below, this difference was largely present already at baseline (Figure S5A).

Consistently, NPHS1 immunoreactivity within glomeruli was lower in ByJcl mice than in AJcl mice (Figure 2C,D), and this difference was likewise already evident at baseline (Figure S5B). Because *Nphs1* encodes nephrin, a key component of the slit diaphragm, the lower NPHS1 signal is consistent with reduced glomerular nephrin immunoreactivity in ByJcl. WT1 and NPHS1 measurements were summarized as per‐mouse means across glomerular cross‐sections or accepted glomerular ROIs, respectively (Figure 2B,D).

**FIGURE 3:**
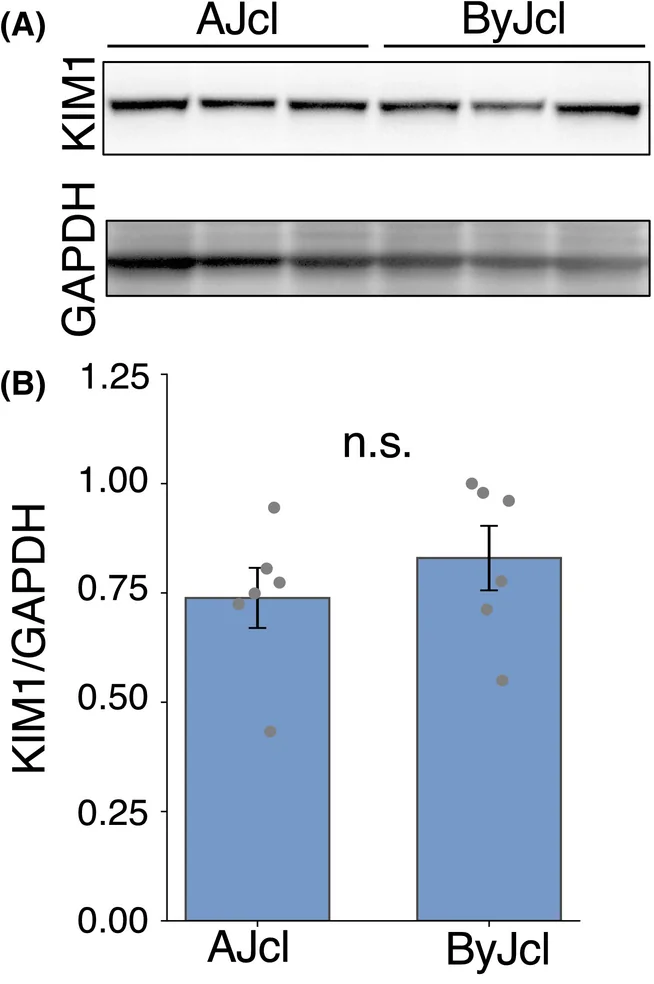
Renal cortical KIM1 protein abundance does not differ between substrains at Day 7. (A) Representative immunoblot of kidney injury molecule‐1 (KIM1) in renal cortex lysates from AJcl and ByJcl mice at D7 after ADR, with GAPDH as a loading control. (B) Densitometric quantification of KIM1 normalized to GAPDH (KIM1/GAPDH) (*n* = 6 per substrain; six mice per substrain were included in the immunoblot analysis). Differences between substrains were evaluated using Welch's two‐tailed *t*‐test. Data are presented as individual values with mean ± SEM. “n.s.” indicates no statistically significant difference.

In contrast, KIM1 protein abundance in renal cortex, assessed by immunoblotting and normalized to GAPDH, did not differ significantly between substrains at Day 7 (Figure 3A,B). The immunoblot analysis included six mice per substrain. Thus, although tubular casts were more frequent in ByJcl mice, KIM1 abundance did not provide evidence for a clear substrain‐dependent difference in overt proximal tubular injury at this early time point.

### 3.3 Baseline differences in podocyte‐associated readouts between substrains

**FIGURE 4:**
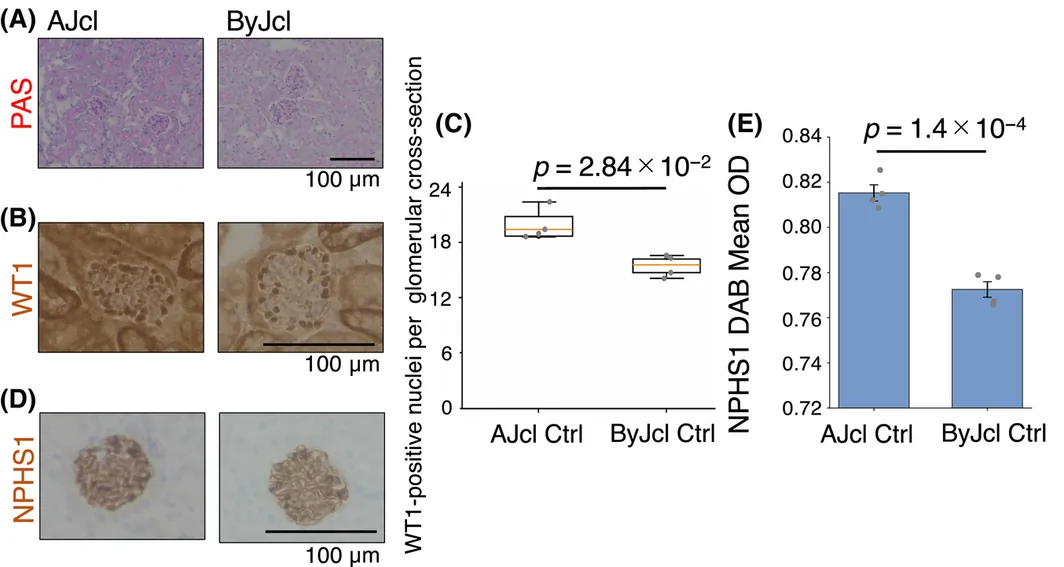
Baseline differences in podocyte‐associated readouts between AJcl and ByJcl mice. (A) Representative PAS‐stained sections of renal cortex from untreated (baseline) BALB/cAJcl (AJcl) and BALB/cByJcl (ByJcl) mice (*n* = 4 per substrain). Scale bar, 100 μm. (B) Representative WT1 immunohistochemistry at baseline (*n* = 4 per substrain). (C) Quantification of WT1‐positive nuclei per glomerular cross‐section at baseline (*n* = 4 per substrain; 30 glomerular cross‐sections per mouse, blinded counting; per‐mouse mean used as the unit of analysis). Differences between substrains were evaluated using the Mann–Whitney *U* test. Data are presented as box‐and‐whisker plots with individual per‐mouse values. (D) Representative nephrin (NPHS1/*Nphs1*) immunohistochemistry at baseline (*n* = 4 per substrain). (E) Quantification of baseline nephrin immunoreactivity (Mean_OD) using the same image‐processing pipeline and sampling strategy described in Methods (*n* = 4 per substrain; all accepted glomeruli analyzed per mouse). Differences between substrains were evaluated using Welch's two‐tailed *t*‐test. Data are presented as individual per‐mouse values with mean ± SEM.

To determine whether substrain‐dependent susceptibility may be rooted in pre‐existing differences, kidneys from untreated (baseline) mice were analyzed. PAS staining revealed no apparent differences in baseline glomerular morphology between AJcl and ByJcl mice (Figure 4A). However, quantitative analysis demonstrated that the number of WT1‐positive nuclei per glomerular cross‐section was significantly higher in AJcl mice than in ByJcl mice at baseline (Figure 4B,C). These findings indicate that ByJcl mice have lower WT1‐positive nuclei counts per glomerular cross‐section prior to ADR exposure, although this measurement cannot distinguish reduced podocyte number from reduced WT1 expression.

In addition, baseline NPHS1 immunoreactivity was lower in ByJcl mice than in AJcl mice (Figure 4D,E). Together, the reduced WT1‐positive nuclei counts and lower NPHS1 staining in ByJcl suggest an attenuated podocyte‐associated state at baseline. These glomerular‐level readouts, summarized using per‐mouse means, support a substrain‐associated difference in podocyte‐associated state at baseline.

### 3.4 Baseline glomerular transcriptomic differences between substrains were limited but included distinct substrain‐associated genes

**FIGURE 5:**
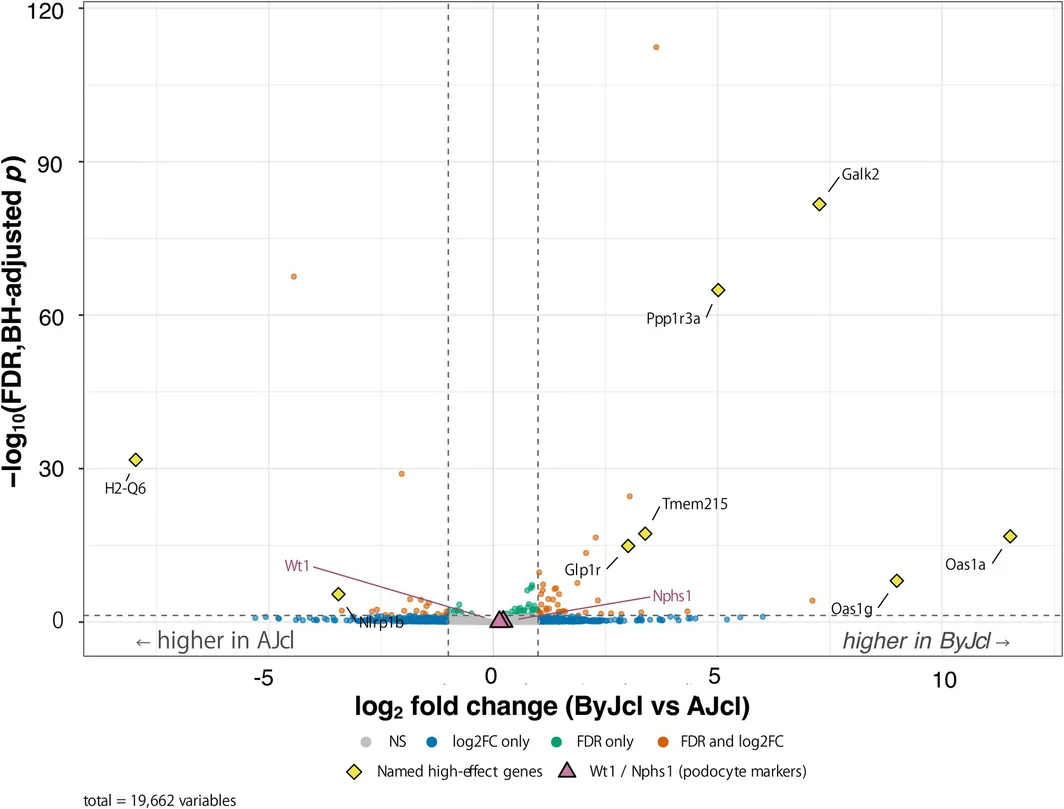
Baseline glomerular transcriptomic differences between AJcl and ByJcl are modest. Differential expression analysis of glomerulus‐enriched RNA‐seq data from untreated BALB/cAJcl (AJcl) and BALB/cByJcl (ByJcl) mice (*n* = 3 per substrain), shown as a volcano plot (ByJcl relative to AJcl; positive log_2_ fold change indicates higher expression in ByJcl). Genes were tested using DESeq2, and significance was defined by Benjamini–Hochberg adjusted *p* value (false discovery rate, FDR) <0.05; dashed lines indicate FDR = 0.05 and |log_2_ fold change| = 1. Of 19 662 tested genes, 103 were significantly different between substrains (FDR <0.05). The podocyte markers Wt1 and Nphs1 are labeled and were not individually differentially expressed at baseline.

To further assess molecular differences at baseline, RNA sequencing was performed on isolated glomeruli from untreated AJcl and ByJcl mice. Differential expression analysis identified 103 of 19 662 tested genes as significantly different between substrains at baseline (FDR <0.05), corresponding to 0.5% of the tested transcriptome. Among these, 64 genes additionally showed a fold change greater than two‐fold (|log_2_FC| >1), indicating that baseline transcriptional divergence was limited on a global scale but included a discrete subset of substrain‐associated transcripts (Figure 5, Table S1).

Several of these genes showed large effect sizes. Among the most divergent were Oas1a (log_2_FC = +11.52, FDR = 1.7 × 10^−17^), H2‐Q6 (−7.96, FDR = 1.9 × 10^−32^), Galk2 (+7.27, FDR = 2.1 × 10^−82^), and Ppp1r3a (+5.01, FDR = 1.3 × 10^−65^), as well as Nlrp1b (−3.45, FDR = 3.5 × 10^−6^), Tmem215 (+3.39, FDR = 5.2 × 10^−18^), and Glp1r (+3.01, FDR = 1.3 × 10^−15^); Tmem215 and Glp1r were higher in ByJcl glomeruli, whereas Nlrp1b was higher in AJcl. Notably, several of the largest baseline differences fall at loci known to be polymorphic among inbred mouse strains, including the Oas1 cluster, the MHC region (H2‐Q6), and Nlrp1b^31^; the interpretation of these genes is considered further in the Discussion.

**FIGURE 6:**
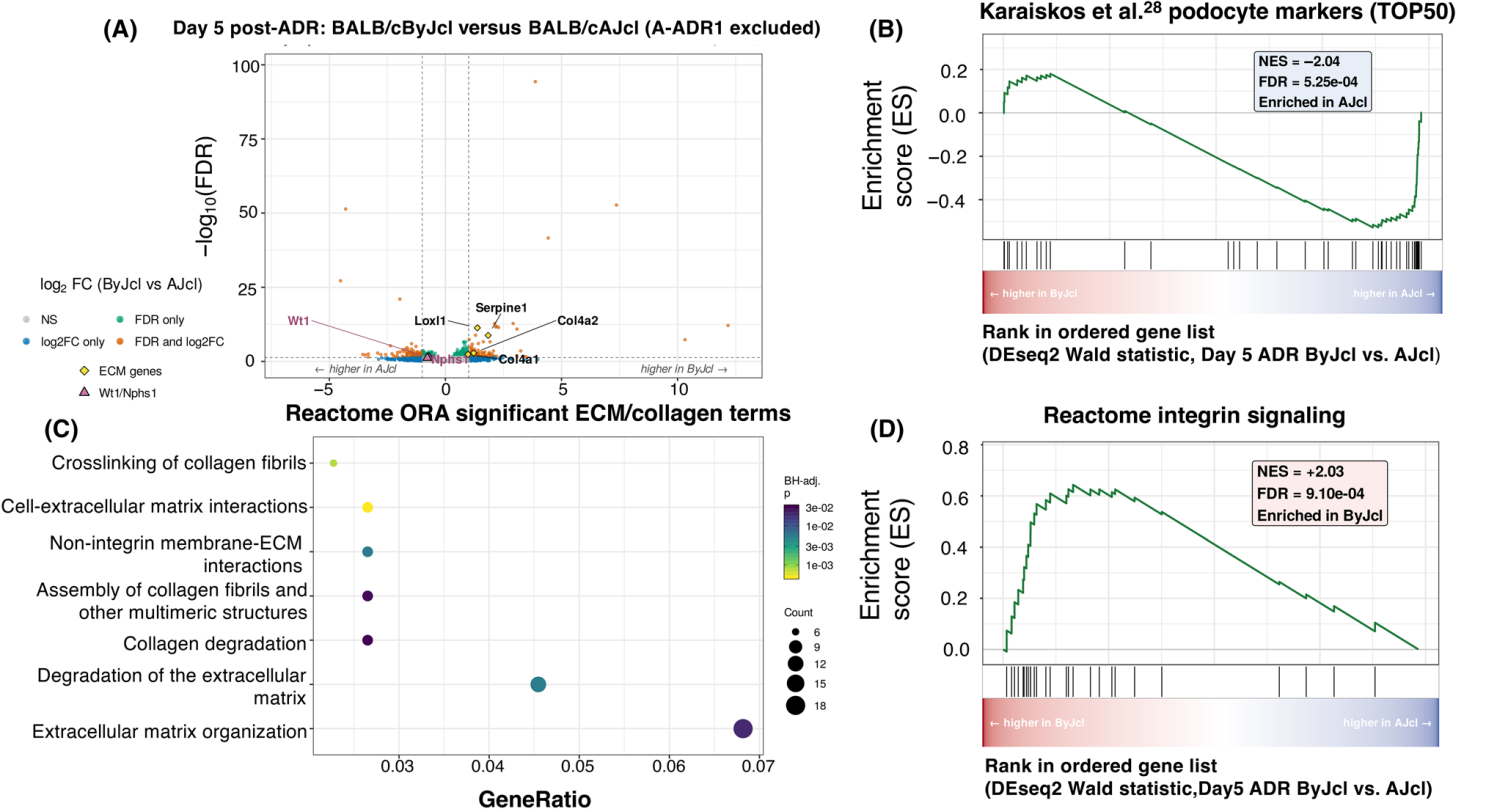
Early glomerular program‐level divergence at Day 5 after ADR: Suppression of podocyte signatures and enrichment of ECM/adhesion pathways in ByJcl. (A) Volcano plot of differential expression analysis of glomerulus‐enriched RNA‐seq at Day 5 (D5) after ADR administration comparing BALB/cByJcl (ByJcl) versus BALB/cAJcl (AJcl) (ByJcl, *n* = 3; AJcl, *n* = 2); positive log_2_ fold change indicates higher expression in ByJcl. Differential expression was tested using DESeq2; Benjamini–Hochberg adjusted *p* values (FDR) are shown (*y*‐axis, −log_10_ FDR). The podocyte markers Wt1 and Nphs1 are labeled. One AJcl sample (A‐ADR1) was excluded due to reduced glomerular purity (see Methods). (B) Preranked gene set enrichment analysis of a single‐cell–derived mouse podocyte marker set (set size = 49)^28^ in ByJcl relative to AJcl at D5 (ByJcl, *n* = 3; AJcl, *n* = 2). Genes were ranked by the DESeq2 Wald statistic; the podocyte program showed significant negative enrichment in ByJcl (NES = −2.04; FDR = 5.3 × 10^−4^, Benjamini–Hochberg across all tested gene sets). Leading‐edge genes included Nphs1, Nphs2, Wt1, Podxl, Synpo, Mafb, and Tcf21. (C) Reactome pathway over‐representation analysis of the DESeq2‐derived differentially expressed genes (546 genes; background = 19 662 tested genes) at D5, highlighting enrichment of extracellular matrix (ECM)– and collagen‐related processes in ByJcl glomeruli (ByJcl, *n* = 3; AJcl, *n* = 2). Dot size indicates the number of genes (Count), and color indicates BH‐adjusted *p* values. Of 734 tested Reactome terms, 19 were significantly enriched (BH‐adjusted *p* < 0.05). (D) Preranked gene set enrichment analysis of the Reactome “Integrin signaling” gene set in ByJcl relative to AJcl at D5 (ByJcl, *n* = 3; AJcl, *n* = 2). The gene set showed significant positive enrichment in ByJcl (NES = +2.03; FDR = 9.1 × 10^−4^, Benjamini–Hochberg across all tested gene sets).

In contrast, canonical podocyte‐associated transcripts were not differentially expressed between substrains at baseline (Wt1: log_2_FC = +0.14, FDR = 0.87; Nphs1: log_2_FC = +0.23, FDR = 0.79); both genes are labeled on the volcano plots (Figures 5 and 6A) to allow direct inspection.

### 3.5 Joint analysis of baseline and ADR‐treated podocyte‐associated readouts

To explicitly determine whether the podocyte‐associated differences observed after ADR reflected ADR‐induced changes or pre‐existing substrain differences, baseline and ADR‐treated groups were analyzed jointly by two‐way ANOVA with substrain and treatment as factors (Figure S5). For WT1‐positive cell counts, there was a strong main effect of substrain (*F*_1,18_ = 113.8, *p* < 0.0001), but no significant treatment effect (*p* = 0.39) and no substrain × treatment interaction (*p* = 0.59). Thus, the lower number of WT1‐positive cells in ByJcl mice primarily reflected a pre‐existing substrain difference rather than a detectable ADR‐induced reduction at Day 7.

For NPHS1 optical density, there was also a strong main effect of substrain (*F*_1,18_ = 59.0, *p* < 0.0001), together with a significant main effect of treatment (*p* = 0.0013), but no substrain × treatment interaction (*p* = 0.82). Tukey's post hoc comparisons showed that NPHS1 was lower in ByJcl than AJcl at both baseline and Day 7 (*p*_adj ≤0.001), and that the baseline‐to‐Day‐7 reduction was statistically significant within AJcl (*p*_adj = 0.048) but not within ByJcl. This asymmetry may partly reflect the lower baseline NPHS1 signal in ByJcl, leaving less detectable room for a further ADR‐associated decrease in this assay. Therefore, the podocyte‐associated phenotype in ByJcl mice is best interpreted as a pre‐existing attenuated podocyte‐associated state that remains evident after ADR exposure, rather than as a substrain‐specific amplification of WT1 or NPHS1 loss at Day 7.

### 3.6 Early transcriptomic divergence in isolated glomeruli at day 5 after ADR

To investigate early molecular responses preceding overt histological divergence, RNA sequencing was performed on isolated glomeruli at Day 5 after ADR administration. Purity assessment based on marker expression confirmed glomerular enrichment; however, one AJcl sample (A‐ADR1) showed reduced glomerular purity (Figure S3) and was excluded from the main analysis to avoid confounding. A sensitivity analysis including this sample is provided in Table S3. In the primary Day 5 ByJcl‐versus‐AJcl comparison, the focal podocyte, ECM, and integrin enrichments reported below were robust to inclusion or exclusion of A‐ADR1. Sensitivity results for the within‐AJcl ADR‐versus‐control comparison are reported separately in Table S3.

Differential expression analysis identified only modest gene‐level differences between substrains at this early time point (Figure 6A). We therefore next applied gene set analyses to assess coordinated shifts in podocyte programs and injury‐response pathways. Using a podocyte marker panel derived from single‐cell RNA sequencing of mouse glomeruli,^28^ preranked gene set enrichment analysis (fgsea v1.24.0; genes ranked by the DESeq2 Wald statistic; FDR by Benjamini–Hochberg across all tested gene sets) revealed significant negative enrichment of the podocyte program in ByJcl relative to AJcl (NES = −2.04, FDR = 5.3 × 10^−4^; leading‐edge genes included Nphs1, Nphs2, Wt1, Podxl, Synpo, Mafb, and Tcf21; Figure 6B). A more stringent podocyte‐exclusive marker set gave a concordant result (NES = −2.22, FDR = 9.3 × 10^−5^; Table S5). Individual podocyte markers showed considerably weaker signal than the coordinated program: Wt1 was marginally significant (log_2_FC = −0.71, FDR = 0.0498) and Nphs1 approached but did not reach significance (log_2_FC = −0.78, FDR = 0.064), both reduced in ByJcl (labeled in Figure 6A). The much stronger gene‐set‐level effect is consistent with a coordinated attenuation of podocyte‐associated transcriptional programs that is not well captured by any single marker gene.

**FIGURE 7:**
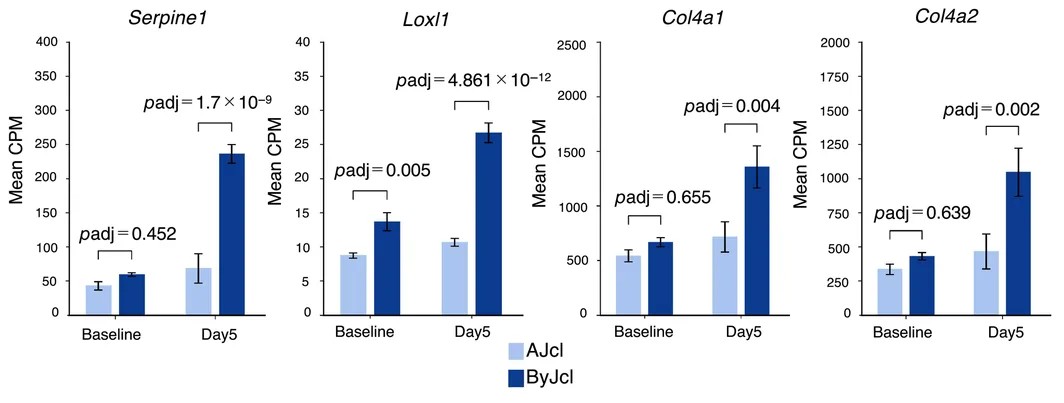
ECM/adhesion‐related transcript expression at baseline and Day 5 after ADR. Expression (counts per million, CPM; mean ± SEM) of the extracellular matrix– and adhesion‐related transcripts *Serpine1*, *Loxl1*, *Col4a1*, and *Col4a2* in BALB/cAJcl (AJcl) and BALB/cByJcl (ByJcl) glomeruli at baseline and at Day 5 after adriamycin (ADR) administration (*n* = 3 per group, except AJcl Day 5, *n* = 2 after exclusion of sample A‐ADR1; see Methods). Numbers displayed above the substrain comparisons in the figure are Benjamini–Hochberg‐adjusted *p* values (FDR) from the final DESeq2 analysis reported in Table S1. At baseline, *Loxl1* was significantly higher in ByJcl (log_2_FC = +0.68, FDR = 0.0052), whereas *Serpine1*, *Col4a1*, and *Col4a2* were not significant (FDR = 0.452, 0.655, and 0.639, respectively). At Day 5, all four transcripts were significantly higher in ByJcl than in AJcl: *Serpine1* (log_2_FC = +1.84, FDR = 1.7 × 10^−9^), *Loxl1* (+1.37, FDR = 4.9 × 10^−12^), *Col4a1* (+0.97, FDR = 4.5 × 10^−3^), and *Col4a2* (+1.22, FDR = 1.5 × 10^−3^). This pattern is consistent with a pre‐existing cell–matrix transcriptional state with additional gene‐level differences evident at Day 5.

In parallel, gene‐set analyses showed that cell–matrix transcriptional programs were already enriched in ByJcl at baseline and remained enriched at Day 5. At Day 5, Reactome pathway over‐representation analysis also identified enrichment of ECM‐and adhesion‐related pathways (Figure 6C).Preranked GSEA showed positive enrichment of Reactome “Extracellular matrix organization” in ByJcl at baseline (NES = +2.28, FDR = 7.0 × 10^−10^) and at Day 5 (NES = +2.19, FDR = 9.7 × 10^−11^). Reactome “Integrin signaling” showed the same pattern (baseline: NES = +1.84, FDR = 0.022; Day 5: NES = +2.03, FDR = 9.1 × 10^−4^; Figure 6D; Table S5). At Day 5, Reactome pathway over‐representation analysis of the 546 DEGs further identified 19 of 734 tested terms as significantly enriched (BH‐adjusted *p* < 0.05), with 16 of the 20 top‐ranked terms related to ECM, collagen, integrin, or adhesion biology. The top term was “Cell–extracellular matrix interactions” (7 genes; BH‐adjusted *p* = 4.8 × 10^−4^), and “Extracellular matrix organization” was also significant (18 genes; BH‐adjusted *p* = 0.020) (Figure 6C). Among the four ECM/adhesion‐related transcripts shown in Figure 7, *Loxl1* was already significantly higher in ByJcl at baseline (log_2_FC = +0.68, FDR = 0.0052), whereas *Serpine1*, *Col4a1*, and *Col4a2* were not significant. At Day 5, all four transcripts were significantly higher in ByJcl than in AJcl: *Serpine1* (log_2_FC = +1.84, FDR = 1.7 × 10^−9^), *Loxl1* (+1.37, FDR = 4.9 × 10^−12^), *Col4a1* (+0.97, FDR = 4.5 × 10^−3^), and *Col4a2* (+1.22, FDR = 1.5 × 10^−3^). Together, these findings support a pre‐existing cell–matrix transcriptional state in ByJcl that persists at Day 5, with additional gene‐level differences evident after ADR.

### 3.7 The ADR responses were broadly concordant in direction across substrains

To determine whether the two substrains mounted qualitatively different responses to ADR, we compared the baseline‐to‐Day 5 change within each substrain. The genome‐wide ADR responses were positively correlated between substrains (Pearson *r* = 0.535, Spearman *ρ* = 0.425; *n* = 19 662). Directional concordance increased with statistical support: 62.8% genome‐wide, 87.0% among genes significant in either substrain (*n* = 2715), and 99.6% among genes significant in both (*n* = 257). Only 24 of 19 662 genes showed a significant substrain × treatment interaction (FDR <0.05; Table S6), and these genes did not form a coherent functional pathway (no significant GO biological‐process enrichment; Reactome enrichment was limited to a single tropomyosin gene pair). Thus, the ADR responses of the two substrains were broadly concordant in direction, particularly among genes with statistical support, while significant substrain × treatment interactions were detected for only a small, functionally dispersed set of genes (Figure S6, Table S6).

## 4 DISCUSSION

In the present study, we demonstrate that two closely related BALB/c substrains, AJcl and ByJcl, exhibit distinct early susceptibility to ADR‐induced nephropathy. ByJcl mice developed more severe albuminuria at Day 7 after ADR administration and showed lower podocyte‐associated readouts, including WT1‐positive nuclei per glomerular cross‐section and NPHS1 immunoreactivity. Notably, these podocyte‐associated differences were already evident at baseline, and no significant substrain × treatment interaction was detected for either readout. Thus, the Day 7 differences are best interpreted as the persistence of a pre‐existing attenuated podocyte‐associated state in ByJcl mice rather than as greater ADR‐induced loss of these markers. These findings suggest that intrinsic substrain‐dependent factors shape the initial cellular context and influence the early trajectory of ADR‐induced injury.

A central observation was that ByJcl mice displayed fewer WT1‐positive nuclei per glomerular cross‐section at baseline than AJcl mice, despite no apparent differences in baseline glomerular morphology by PAS staining. Because WT1 is widely used as a marker of differentiated podocytes,^32^ reduced WT1‐positive cell counts may reflect a lower podocyte endowment, altered WT1 expression per cell, and/or subtle differences in podocyte identity prior to injury. Consistently, baseline NPHS1 immunoreactivity was also lower in ByJcl glomeruli. Together, these findings support the notion that ByJcl kidneys begin from a comparatively attenuated podocyte‐associated state, which may render the filtration barrier more vulnerable to toxic stress.

With this caveat, two genes are notable for their effect size and expression level. Tmem215 was expressed at higher levels in ByJcl glomeruli (log_2_FC = +3.39, FDR = 5.2 × 10^−18^). TMEM215 is an endoplasmic reticulum–resident transmembrane protein expressed in endothelial cells, where it supports endothelial survival and angiogenic responses.^33^ Its higher baseline expression in ByJcl may therefore reflect subtle differences in the glomerular endothelial compartment. We note, however, that the direction of this difference is not readily reconciled with greater susceptibility: if TMEM215 promotes endothelial survival, its higher expression in the more susceptible substrain would not, by itself, predict greater injury.

Glp1r was likewise expressed at higher levels in ByJcl glomeruli (log_2_FC = +3.01, FDR = 1.3 × 10^−15^). GLP‐1 receptor signaling has well‐documented kidney‐protective effects, including attenuation of oxidative stress and inflammatory injury.^34^ As with Tmem215, the higher baseline expression of a putatively protective receptor in the more susceptible substrain is not readily interpreted as a susceptibility mechanism, and may instead represent a compensatory or context‐dependent state. An additional consideration is cellular composition: the renal localization of GLP‐1R remains contested, with studies using validated antibodies, in situ hybridization, and single‐cell transcriptomics localizing GLP‐1R principally to juxtaglomerular cells, preglomerular vascular smooth muscle, and endothelial cells rather than to podocytes.^35^ Because juxtaglomerular and vascular structures co‐purify with glomeruli in bead‐perfusion preparations, a substrain difference in Glp1r signal in glomerulus‐enriched bulk RNA‐seq may reflect the relative contribution of these compartments rather than a podocyte‐intrinsic difference. Nlrp1b, an inflammasome sensor,^31^ was higher in AJcl glomeruli; given the polymorphic nature of this locus noted above, we do not interpret this difference as establishing a substrain difference in inflammasome activity, and its functional consequence, if any, remains to be determined.

Taken together, baseline substrain variation was not defined by broad remodeling of the canonical podocyte identity program: Wt1 and Nphs1 were not significantly different at the mRNA level (FDR = 0.87 and 0.79, respectively), despite the reduced WT1‐positive nuclei counts and NPHS1 immunoreactivity observed histologically. This discordance between protein‐level readouts and bulk mRNA profiles is consistent with several non‐mutually exclusive scenarios, including post‐transcriptional regulation, altered protein stability or localization, subtle shifts in differentiation state, and/or modest differences in cellular composition that are not readily captured by bulk glomerular RNA‐seq.^36^ Rather than identifying a single causal transcript, the baseline data indicate that AJcl and ByJcl glomeruli differ in a small number of discrete molecular features—several of which are most parsimoniously attributed to substrain genetic background—while the substrain difference relevant to ADR susceptibility is more evident in the protein‐level podocyte‐associated readouts and in a baseline ECM/integrin transcriptional state that remained enriched at Day 5.

Following ADR administration, the baseline podocyte‐associated disparities remained evident in ByJcl mice and were accompanied by a more pronounced early functional phenotype. At Day 7, ByJcl mice exhibited higher albuminuria and lower WT1‐positive nuclei per glomerular cross‐section and NPHS1 immunoreactivity than AJcl mice. However, no significant substrain × treatment interaction was detected for either podocyte‐associated readout, indicating that these Day 7 differences largely reflected pre‐existing substrain differences rather than greater ADR‐induced decreases in ByJcl mice.

In contrast, KIM1 abundance in the renal cortex did not differ between substrains at this time point. Thus, although tubular casts were more frequent in ByJcl mice, KIM1 did not provide evidence of a clear substrain‐dependent difference in overt proximal tubular injury. The tubular casts may represent proteinuria‐associated tubular changes accompanying the more severe glomerular functional phenotype, although a contribution from tubular injury cannot be excluded.

Additional insight into early divergence was provided by transcriptomic profiling of isolated glomeruli at Day 5 after ADR administration. Because gene‐level differential expression between substrains was modest at this time point, we applied gene set analyses to assess coordinated program‐level shifts. Preranked GSEA revealed significant negative enrichment of podocyte‐associated gene signatures in ByJcl relative to AJcl, indicating coordinated attenuation of podocyte transcriptional programs during early injury.

This program‐level suppression is notable because it aligns with the broader “podocyte depletion/podocytopathy” framework, in which podocytes are highly specialized cells with limited capacity for replacement, and loss or dysfunction of a critical fraction of podocytes is thought to drive proteinuria and subsequent glomerulosclerosis.^37^ In this framework, baseline podocyte endowment—often quantified relative to glomerular size as podocyte density or related “podometrics”—can shape susceptibility and the kinetics of disease progression, such that a lower “reserve” may be more readily pushed past a functional threshold when injury supervenes.^38^ In this context, the ByJcl pattern—baseline reduction in podocyte‐associated immunoreactivity together with early, coordinated suppression of podocyte gene programs—is consistent with an attenuated podocyte‐associated state and/or earlier decompensation of podocyte programs under stress. Importantly, the fact that the effect is most evident at the gene‐set level (rather than as large single‐gene changes in canonical markers) supports the idea that early divergence may reflect a distributed shift across multiple components of the podocyte structural and slit diaphragm programs, which could precede overt morphological change and contribute to subsequent amplification of albuminuria.

In parallel, GSEA showed that ECM organization and integrin signaling were enriched in ByJcl at baseline and remained enriched at Day 5.^39^ This pattern supports a pre‐existing cell–matrix transcriptional state that persists after ADR exposure, rather than pathway activation induced specifically by ADR. *Serpine1* (PAI‐1) is a central regulator of pericellular proteolysis via inhibition of plasminogen activators and has been repeatedly linked to matrix accumulation and renal fibrogenic remodeling across experimental and clinical contexts.^40^ At the individual‐gene level, *Serpine1* was significantly higher in ByJcl at Day 5; because PAI‐1 inhibits plasminogen activators, this increase may reflect reduced protease‐driven ECM turnover; however, our data show only its transcriptional upregulation and do not establish a functional effect on matrix turnover in this model. Importantly, PAI‐1 biology can intersect with podocyte‐relevant adhesion signaling through the uPA/uPAR axis and related pathways that have been implicated in proteinuric kidney disease and discussed in connection with integrin‐dependent mechanisms.^41^

At Day 5, increased expression of *Col4a1* and *Col4a2* suggests remodeling of basement membrane architecture, as type IV collagen networks constitute core structural scaffolds of the GBM and shape ligand availability for integrin‐based adhesion.^41^ Even modest shifts in basement membrane collagen production—particularly if spatially localized to the GBM—might influence integrin engagement and adhesion dynamics in podocytes during a phase when podocyte‐associated programs are already relatively attenuated, although this was not directly assessed. In parallel, *Loxl1* was already higher in ByJcl at baseline and remained elevated at Day 5; this is notable because lysyl oxidase family activity catalyzes oxidative cross‐linking that stabilizes ECM architecture, increases resistance to degradation, and contributes to fibrogenic remodeling through matrix stabilization and altered tissue mechanics.^42^ These three transcripts—*Serpine1*, the type IV collagens, and *Loxl1*—are each individually associated with ECM retention and remodeling in other systems, and their expression pattern in ByJcl is consistent with, but does not by itself demonstrate, a persistent shift toward ECM retention at the cell–matrix interface.

These persistent ECM/integrin signatures are compatible with at least two non‐mutually exclusive scenarios. One possibility is that a constitutive substrain‐specific cell–matrix state provides compensatory stabilization of podocyte–GBM attachment and remains evident during early injury. Alternatively, the same pre‐existing state may favor matrix retention, cross‐linking, altered mechanotransduction, and integrin signaling that predispose ByJcl glomeruli to the more severe functional phenotype observed at Day 7. Discriminating adaptive stabilization from maladaptive remodeling will require spatial validation,^40^, ^41^, ^42^, ^43^ integrin activation‐state readouts, and time‐course studies extending beyond the early phase examined here.

Biologically, integrin‐dependent adhesion is fundamental for podocyte attachment to the GBM, and podocyte integrin pathways are tightly coupled to actin cytoskeletal organization and slit diaphragm stability. Genetic or functional perturbation of podocyte integrin signaling compromises filtration barrier integrity and can induce proteinuria.^39^ More broadly, integrin‐dependent signaling regulates glomerular cell behavior and matrix organization in health and disease, including bidirectional feedback between matrix composition/structure and cell‐state programs.^14^, ^44^ In this context, enrichment of ECM‐ and integrin‐associated signatures in ByJcl at both baseline and Day 5 is best interpreted as a persistent substrain‐associated cell–matrix state that coexists at Day 5 with coordinated attenuation of podocyte programs, rather than as a response induced specifically by ADR. While ECM accumulation is classically associated with progressive glomerulosclerosis and fibrosis, ECM remodeling can also be triggered early after injury and may precede overt histological change.^45^ Thus, persistence of the ByJcl signature after ADR may either stabilize adhesion or contribute to later maladaptation, depending on its spatial distribution, magnitude, and downstream functional effects.

Collectively, our findings highlight the importance of substrain background in experimental nephropathy models. Even within the BALB/c lineage, subtle divergence between substrains can translate into meaningful differences in baseline podocyte‐associated features, persistent cell–matrix states, and early stress responsiveness, potentially affecting reproducibility across laboratories. These results underscore the need for careful documentation of substrain origin and motivate further work to identify the genetic and molecular determinants that shape baseline podocyte‐associated features and early injury trajectories.

Our findings also have implications for the interpretation of previous adriamycin nephropathy studies. BALB/c mice are widely used in this model, but substrain information is frequently unreported. Because BALB/cAJcl and BALB/cByJcl differed substantially in baseline podocyte‐associated readouts and in the magnitude of ADR‐induced albuminuria, results obtained from mice described only as “BALB/c” may depend on which substrain was used. Unreported substrain composition could therefore contribute to variability between studies and complicate direct cross‐study comparison. We suggest that substrain identity be explicitly reported in future adriamycin nephropathy studies, and that comparisons between earlier reports be interpreted with substrain background in mind.

Several limitations should be acknowledged. First, reduced WT1‐positive nuclei counts per glomerular cross‐section do not definitively distinguish decreased podocyte number from altered WT1 expression per cell. Similarly, reduced NPHS1 immunoreactivity may reflect changes in expression level, redistribution of nephrin protein, or early dedifferentiation rather than outright podocyte loss. Second, bulk glomerular RNA‐seq cannot fully exclude subtle differences in cellular composition, and isolated glomeruli may retain variable contributions from non‐podocyte compartments (as suggested by sample‐level purity variation addressed by sensitivity analysis). Third, because the AJcl Day 5 group contained only two samples after quality‐control exclusion, the interaction analysis had limited power to detect modest substrain‐specific differences in the ADR response. Future studies using quantitative podocyte counting strategies, ultrastructural analyses, and cell‐type–resolved transcriptomic approaches will help refine the cellular basis of substrain‐specific divergence and clarify whether the persistent ECM/integrin state is adaptive or maladaptive and how it changes after ADR exposure.

In summary, this study supports a model in which intrinsic baseline differences in podocyte‐associated features between BALB/c substrains are associated with early responses to ADR‐induced injury. ByJcl mice exhibit lower baseline WT1‐positive cell counts and diminished NPHS1 immunoreactivity—interpreted together as an attenuated podocyte‐associated state rather than a definitive reduction in podocyte number—followed by greater albuminuria after ADR exposure. Glomerular transcriptomics further indicated that ECM organization and integrin signaling were enriched in ByJcl at baseline and remained enriched at Day 5, when coordinated suppression of podocyte‐associated programs was also evident. These findings highlight the potential role of baseline cellular context, persistent cell–matrix transcriptional states, and early podocyte‐program changes in the trajectory of toxin‐induced glomerular injury, and underscore the importance of documenting substrain origin for reproducibility.

## AUTHOR CONTRIBUTIONS

**Ryuya Nakagawa:** Investigation; validation; visualization; writing – original draft. **Masaki Watanabe:** Conceptualization; investigation; writing – original draft; validation; visualization; data curation. **Nobuya Sasaki:** Conceptualization; funding acquisition; writing – review and editing; project administration; resources; supervision. **Momoka Hiratsuka:** Investigation; visualization. **Koyuki Kawamoto:** Investigation; visualization. **Takeru Sasaki:** Investigation; validation; data curation.

## FUNDING INFORMATION

This research received no specific grant from any funding agency in the public, commercial, or not‐for‐profit sectors.

## CONFLICT OF INTEREST STATEMENT

The authors declare no conflict of interest.

## ETHICS STATEMENT

All procedures followed the Regulations for Animal Experiments of Kitasato University. The protocol was reviewed by the Kitasato University Institutional Animal Care and Use Committee and approved by the University President (Approval ID: 24–094).

## Supporting information


**Figure S1.** Representative SDS‐PAGE for semi‐quantitative urinary albumin assessment. Representative Coomassie Brilliant Blue–stained SDS‐PAGE gel of urine samples collected at Day 7 after adriamycin administration from BALB/cAJcl (AJcl) and BALB/cByJcl (ByJcl) mice (*n* = 7 per substrain). Bovine serum albumin (BSA; 2.5 μg) was electrophoresed in parallel as a positive control, and the urinary albumin band (~66 kDa) was quantified by densitometry using Fiji/ImageJ. Band intensity was normalized to the urinary creatinine concentration of the same sample to derive the semi‐quantitative albumin‐to‐creatinine index (Alb/Cre; arbitrary units per mg/dL creatinine). Images were processed uniformly across the entire gel, with no lane‐specific adjustment.
**Figure S2.** Representative automated glomerular ROI detections for NPHS1 quantification. Representative brightfield images of NPHS1 (nephrin) immunohistochemistry with the corresponding automatically detected accepted glomerular regions of interest (ROIs) overlaid, illustrating the image‐analysis pipeline described in Methods (*n* = 4 baseline and *n* = 7 ADR mice per substrain). Candidate glomeruli were detected by Otsu thresholding of a Gaussian‐smoothed DAB optical‐density image, followed by morphological filtering and removal of border‐touching objects. For each accepted glomerulus, a convex‐hull ROI was generated and the mean DAB optical density (Mean_OD) was calculated. All accepted glomeruli per sample were analyzed, and the per‐mouse mean Mean_OD was used as the unit of analysis.
**Figure S3.** Glomerular purity assessment across all RNA‐seq samples. Podocyte‐to‐tubular marker‐sum ratio (log scale) for all 12 glomerulus‐enriched RNA‐seq samples (four groups: A‐Ctrl, B‐Ctrl, A‐ADR, B‐ADR; *n* = 3 each), calculated as the summed CPM of podocyte markers divided by the summed CPM of renal‐tubular markers (*Slc12a1*, *Umod*, *Aqp2*, *Lrp2*, *Slc34a1*, *Aqp1*). Among all 12 samples, A‐ADR1 showed the lowest ratio (2.51); the next‐lowest sample overall was A‐Ctrl3 (4.50; approximately 1.8‐fold higher), and the next‐lowest ADR sample was approximately 2.8‐fold higher. Together with elevated expression of multiple renal‐tubular markers (lower panel), this indicated reduced glomerular purity; A‐ADR1 was therefore excluded from the primary Day 5 analysis. Baseline (control) samples formed a continuous distribution on this metric and were all retained. Gray band, range of retained samples; dashed line, their median. Lower panel: CPM (log₁₀) of the six renal‐tubular markers across all samples, with A‐ADR1 outlined.
**Figure S4.** Body‐weight time course in ADR‐treated AJcl and ByJcl mice. Absolute body weight (g) in adriamycin (ADR)‐treated BALB/cAJcl (AJcl) and BALB/cByJcl (ByJcl) mice is plotted for each day through Day 7 (*n* = 7 per substrain). Values are shown as mean ± SEM; individual replicates are not shown. The statistical comparison was performed on percent change from Day 0 rather than on the absolute weights plotted: ADR‐treated AJcl mice lost more weight than ByJcl mice (mean nadir −13.8% vs. −9.8%; Mann–Whitney *U* test, *p* = 0.017). Two of seven AJcl mice transiently exceeded 15% body‐weight loss, whereas no ByJcl mouse did; however, all animals remained below 20% body‐weight loss, and none met the humane endpoint or was lost or excluded (see Methods). Thus, ByJcl mice exhibited less body‐weight loss despite greater renal injury, indicating that the renal phenotype was not accompanied by greater systemic weight loss.
**Figure S5.** Two‐way ANOVA of WT1 and NPHS1 at baseline and Day 7. WT1‐positive nuclei counts and NPHS1 optical density in BALB/cAJcl (AJcl) and BALB/cByJcl (ByJcl) glomeruli at baseline and at Day 7 after adriamycin (ADR), analyzed jointly by two‐way ANOVA (substrain × treatment) with Tukey's HSD post hoc test. Bars show mean ± SEM with per‐mouse values overlaid (*n* = 4 baseline and *n* = 7 ADR per substrain). (A) WT1‐positive nuclei per glomerular cross‐section showed a strong main effect of substrain (*F*_1,18_ = 113.8, *p* < 0.0001), with no significant effect of treatment (*p* = 0.39) and no substrain × treatment interaction (*p* = 0.59); baseline and ADR groups of the same substrain did not differ (Tukey's HSD, AJcl *p*_adj = 0.995, ByJcl *p*_adj = 0.75). (B) NPHS1 mean optical density showed a strong main effect of substrain (*F*_1,18_ = 59.0, *p* < 0.0001) and a significant effect of treatment (*p* = 0.0013), with no interaction (*p* = 0.82); the baseline‐to‐Day‐7 reduction was significant within AJcl (Tukey's HSD, *p*_adj = 0.048) but not within ByJcl. Together these analyses indicate that the lower WT1 and NPHS1 signals in ByJcl are primarily pre‐existing substrain differences already present at baseline rather than substrain‐specific amplification of ADR‐induced reductions.
**Figure S6.** Cross‐substrain comparison of the ADR transcriptional response. Scatter plot comparing the ADR‐induced transcriptional response (baseline‐to‐Day 5 log_2_ fold change) between substrains, with the AJcl response on the *x*‐axis and the ByJcl response on the *y*‐axis (*n* = 19 662 tested genes). The underlying within‐substrain contrasts included *n* = 3 baseline samples per substrain, *n* = 3 ByJcl Day 5 samples, and *n* = 2 AJcl Day 5 samples after exclusion of A‐ADR1. The two substrains' responses were positively correlated (Pearson *r* = 0.535, Spearman *ρ* = 0.425), and directional concordance increased with statistical support (62.8% genome‐wide; 87.0% among genes significant in either substrain, *n* = 2715; 99.6% among genes significant in both, *n* = 257). Genes showing a significant ADR response in either substrain (FDR <0.05 in the AJcl or ByJcl within‐substrain comparison; *n* = 2715) are highlighted, whereas genes that were not significant in either substrain are shown in gray. Separately, 24 of 19 662 genes showed a significant substrain × treatment interaction (FDR <0.05; Table S6); these interaction genes did not form a coherent functional pathway (no significant GO biological‐process enrichment; Reactome enrichment was limited to a single tropomyosin gene pair). Together, these results indicate that the ADR responses of the two substrains were broadly concordant in direction, with substrain‐dependent differences confined to a small, functionally dispersed set of genes.

**Table S1.** Differential expression results for all comparisons. ESeq2 differential expression results (glomerulus‐enriched RNA‐seq) for all substrain and treatment contrasts, including baseline (ByJcl vs. AJcl), Day 5 after ADR (ByJcl vs. AJcl), and the ADR response within each substrain (Day 5 vs. baseline). For each gene, the base mean, log_2_ fold change, standard error, Wald statistic, nominal *p* value, and Benjamini–Hochberg adjusted *p* value (FDR) are reported. All contrasts were computed on the same 19 662 tested genes; positive log_2_ fold change indicates higher expression in ByJcl (for substrain contrasts) or at Day 5 (for within‐substrain ADR contrasts).

**Table S2.** Podocyte marker gene sets. Podocyte marker gene sets used for preranked gene set enrichment analysis in Figure 6B, derived from single‐cell RNA sequencing of the mouse glomerulus.^28^ The original TOP50 list contained 50 marker genes, of which 49 were detected in the count matrix and included in the GSEA; Sept11 was not detected and was therefore excluded from the analysis. The more stringent podocyte‐exclusive confirmatory set contained 12 genes, all of which were detected and included in the GSEA. Eleven of the 12 podocyte‐exclusive genes overlapped the TOP50 marker list; Ptpro was included only in the podocyte‐exclusive set.

**Table S3.** Sensitivity analysis for inclusion or exclusion of sample A‐ADR1. Include/exclude sensitivity analysis for six focal gene sets across the four primary comparisons. A‐ADR1 status was applicable to the Day 5 ByJcl‐versus‐AJcl and AJcl ADR‐versus‐control comparisons; the baseline ByJcl‐versus‐AJcl and ByJcl ADR‐versus‐control comparisons are included for completeness. For each focal gene set, the normalized enrichment score (NES), nominal *p* value, joint Benjamini–Hochberg‐adjusted FDR, gene‐set size, genome‐wide rank, and total number of tested gene sets are reported. In the primary Day 5 ByJcl‐versus‐AJcl comparison, the focal podocyte, ECM, and integrin enrichments were robust to inclusion or exclusion of A‐ADR1. Results for the within‐AJcl ADR‐versus‐control comparison should be interpreted separately. Results are also shown for the podocyte‐aging gene set, which was sensitive to A‐ADR1 inclusion.

**Table S4.** Complete Day‐5 GSEA and over‐representation analysis results. The workbook comprises two worksheets. Table S4A reports the complete preranked gene set enrichment analysis results at Day 5 (ByJcl vs. AJcl; A‐ADR1 excluded), obtained using fgsea with genes ranked by the DESeq2 Wald statistic, for all 1293 tested gene sets, including Reactome and custom podocyte gene sets. Table S4B reports the complete ReactomePA over‐representation analysis results for all 734 tested Reactome terms. Normalized enrichment scores or gene counts, nominal *p* values, Benjamini–Hochberg‐adjusted *p* values/FDR, and other relevant analysis outputs are reported.

**Table S5.** Focal gene set enrichment results. Focal results for the podocyte‐exclusive marker set, Reactome “Extracellular matrix organization,” and Reactome “Integrin signaling” across the four primary comparisons: baseline ByJcl vs. AJcl, Day 5 ByJcl vs. AJcl, AJcl ADR vs. control, and ByJcl ADR vs. control. For each comparison, the normalized enrichment score (NES), nominal *p* value, joint Benjamini–Hochberg‐adjusted FDR, gene‐set size, genome‐wide rank, and total number of tested gene sets are reported; the Day 5 AJcl group excludes sample A‐ADR1.

**Table S6.** Genes with a significant substrain × treatment interaction. The 24 genes showing a significant substrain × treatment interaction (FDR <0.05) in the analysis of the ADR transcriptional response (Figure S6). For each gene, the interaction log2 fold change, Wald statistic, and adjusted *p* value are reported, together with the within‐substrain ADR‐response estimates and the baseline substrain contrast. These genes did not form a coherent functional pathway (no significant GO biological‐process enrichment; Reactome enrichment was limited to a single tropomyosin gene pair).

## Data Availability

The RNA‐seq data generated in this study have been deposited in the NCBI Gene Expression Omnibus (GEO) under accession number GSE320498. The analysis scripts used in this study are publicly available in Zenodo (doi: 10.5281/zenodo.21128268). Other data supporting the findings of this study are available from the corresponding author upon reasonable request.
