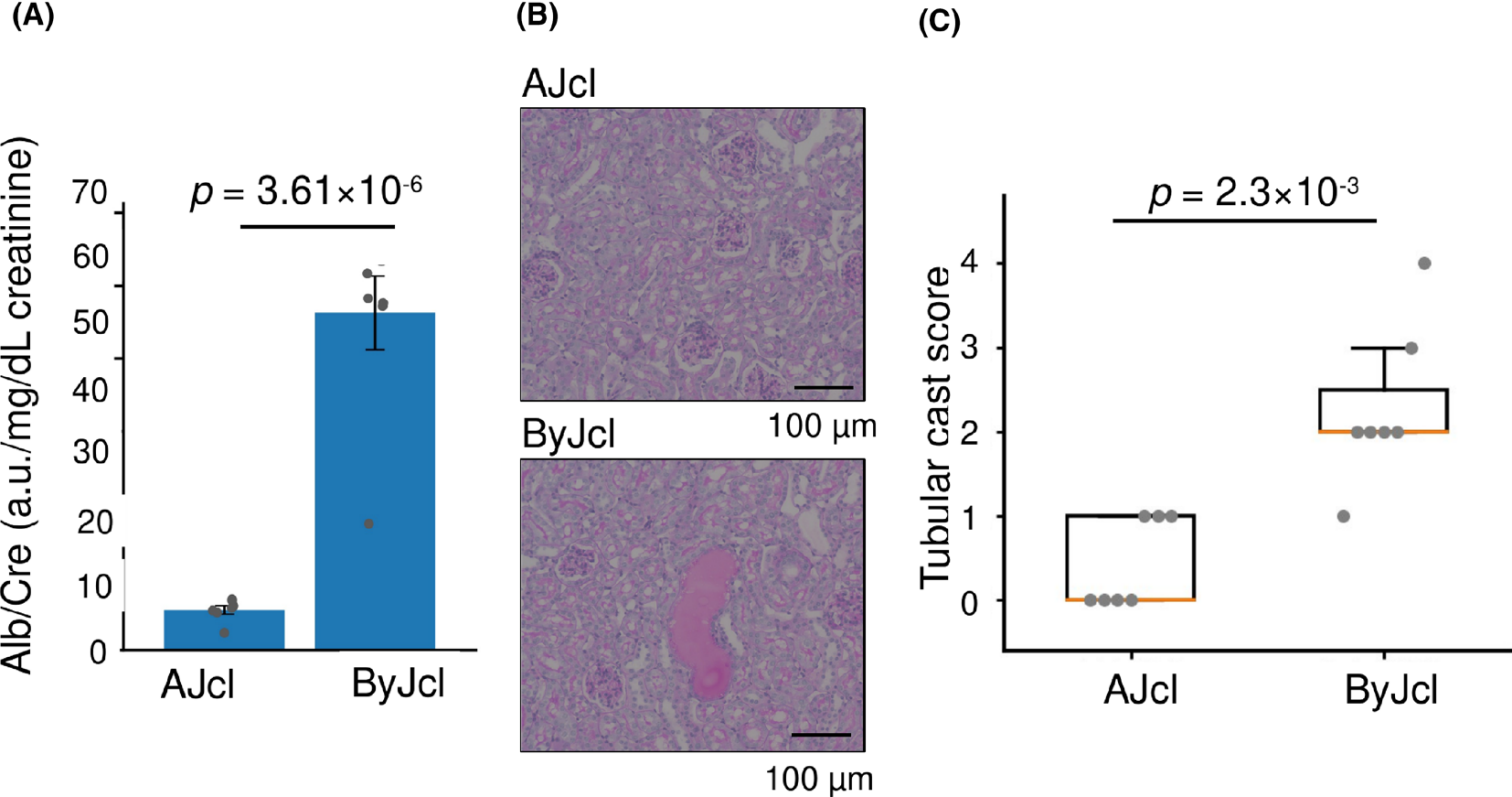# Correction to “Baseline podocyte‐associated state and persistent cell–matrix transcriptional programs are associated with BALB/c substrain differences in adriamycin nephropathy”

**DOI:** 10.1002/ame2.70320

**Published:** 2026-10-16

Nakagawa R , Watanabe M , Kawamoto K , Hiratsuka M , Sasaki T , Sasaki N . Baseline podocyte‐associated state and persistent cell–matrix transcriptional programs are associated with BALB/c substrain differences in adriamycin nephropathy. Anim Models Exp Med. 2026; 9(9): 1729‐1742. doi:10.1002/ame2.70281 PMC1350184442635315

In Figure 1B, the label “AJcl” was inadvertently retained within the representative periodic acid–Schiff (PAS)‐stained image for the ByJcl group during figure preparation. The corrected Figure 1 is provided below, with the unintended label removed. No other elements of Figure 1 have been changed. This correction does not affect the data, statistical analyses, results, interpretation, or conclusions of the article.